# Exploring the Association Between Pulmonary Hypertension and Cancer: A Systematic Review and Meta-Analysis

**DOI:** 10.3390/biomedicines14040876

**Published:** 2026-04-11

**Authors:** Filippo Catalani, Arianna Pannunzio, Emanuele Valeriani, Walter Ageno, Pasquale Pignatelli, Sandor Györik

**Affiliations:** 1 Department of Internal Medicine, Regional Hospital of Bellinzona e Valli, Ente Ospedaliero Cantonale (EOC), 6500 Bellinzona, Switzerland; sandor.gyoerik-lora@eoc.ch; 2 Department of General Surgery, Surgical Specialty and Anesthesiology, Sapienza University of Rome, 00185 Rome, Italy; arianna.pannunzio@uniroma1.it (A.P.); emanuele.valeriani@uniroma1.it (E.V.); pasquale.pignatelli@uniroma1.it (P.P.); 3 Department of Internal Medicine, Endocrino-Metabolic Sciences, and Infectious Disease, Azienda Ospedaliero-Universitaria Policlinico Umberto I, 00161 Rome, Italy; 4 Department of Medicine, University of Padua, 35122 Padua, Italy; walter.ageno@unipd.it

**Keywords:** pulmonary hypertension, PH, chronic thromboembolic pulmonary hypertension, CTEPH, pulmonary circulation, cancer, malignancy, myeloproliferative neoplasm, MPN, mortality

## Abstract

**Background:** Cancer and pulmonary circulation disorders represent increasingly intersecting clinical entities. The prevalence of malignancy in patients with pulmonary hypertension (PH), particularly those with chronic thromboembolic pulmonary hypertension (CTEPH), is higher than in the general population. Moreover, cancer and antineoplastic therapies have been implicated in the development of PH through multiple mechanisms. **Methods:** We performed a systematic review and meta-analysis of the literature focusing on the prevalence of cancer in patients with PH. Mortality incidence and mortality risk were also evaluated for patients with PH with or without cancer. Specific sub-analyses for patients with CTEPH were also performed. Finally, we evaluated the prevalence of PH and its risk of mortality in patients with cancer. **Results:** Overall, 12 studies including 4402 patients were selected in the quantitative analysis. All the included studies had an observational design. The prevalence of cancer in patients with any PH group was 13% (95% CI: 11–16%); mortality incidence in patients with any PH group and cancer was 41% (95% CI: 26–58%), compared to 10% (95% CI: 1–48%) in those without cancer. The association was even stronger when considering only patients with CTEPH, with a mortality incidence of 4% (95% CI: 2–9%) in those without cancer compared to 19% (95% CI: 8–37%) in patients with cancer (*p* for difference: <0.01). Finally, prevalence of any PH group in patients with cancer was 22% (95% CI: 15–31%). **Conclusions:** We observed a possible correlation between PH and cancer, with a significant impact on mortality in patients with PH, particularly those with CTEPH. This association suggests the need for a close clinical surveillance for early detection of cancer and PH.

## 1. Introduction

Pulmonary hypertension (PH), with its heterogeneous clinical presentations, can be suspected by a peak tricuspid regurgitant velocity (TRV) > 3.4 m/s in echocardiography and is defined by a mean pulmonary arterial pressure (mPAP) > 20 mmHg by right heart catheterization (RHC). Its prevalence is estimated to be around 1% of the global population, even more in individuals aged over 65 years, due to the growing incidence of cardiac and pulmonary causes of PH [1].

PH can be divided into groups of pre-capillary, post-capillary, or combined pre- and post- capillary forms, according to the underlying pathophysiological mechanisms, clinical presentation, hemodynamic characteristics, associated diseases, and management. This enables classification into five distinct subgroups: group 1. Pulmonary arterial hypertension (PAH); group 2. PH associated with left heart disease (LHD); group 3. PH associated with lung diseases and/or hypoxia; group 4. PH associated with pulmonary artery obstruction (namely, chronic thromboembolic pulmonary hypertension, CTEPH); group 5. PH with unclear or multifactorial mechanisms [2].

The link between PH and both solid and hematological malignancies (and their respective treatments) has steadily grown in relevance in the last years [3,4,5,6]. Some observational studies showed a significantly increased incidence of malignancies (up to 14.5%; 95% CI: 10.7–18.2%) in patients with pre-capillary PH [7]; this association poses challenges to management and adversely affects the outcomes. This was particularly relevant for patients with CTEPH, with a high prevalence of malignancies and mortality driven more by this association than by PH itself in some studies, with a reported HR of 4.83 (95% CI: 2.27–10.27) for patients with CTEPH and a history of cancer compared to those without [8,9,10].

The pathophysiology of cancer-associated PH is complex, heterogenous, probably underreported, and not fully clarified [11]. In solid tumors, the very well-established association between thromboembolism and cancer represents one possible explanation. However, several less recognized mechanisms may also contribute to the development of PH in this population, including tumor invasion (i.e., angiosarcoma), vascular compression, tumor-related macro- and micro-emboli, tumor thrombotic microangiopathy, which shares pathophysiological similarities with PAH, and pulmonary veno-occlusive disease (PVOD) [12]. In a French registry, PH was associated with a prevalence of 1.3% of myeloproliferative neoplasms (MPNs), one third group 4 and two thirds group 5, and the long-term outcomes were poorer in these patients [13]. Cancer treatment itself can contribute to PH development. With different therapies, all PH subgroups have been described: the high incidence of PH with dasatinib, for example, is emblematic for the tyrosine kinase inhibitors (TKIs) group [14].

Accordingly, increasing research interest has focused on investigating the relationship between malignancies and the pulmonary vasculature, trying to elucidate potential associations among cancer, antineoplastic therapies and PH; nevertheless, the evidence to date remains limited to observational studies, often leading to discordant results. Thus, the aim of this systematic review and meta-analysis was to provide an up-to-date, comprehensive estimate of the association between PH and cancer by pooling the results of the available studies in the field. The main focuses were to assess the prevalence of cancer in patients with PH, the prevalence of PH in patients with cancer (particularly hematological malignancies), and their impact on patients’ mortality.

## 2. Materials and Methods

We conducted a systematic review and meta-analysis according to the Preferred Reporting Items for Systematic reviews and Meta-Analysis (PRISMA) guidelines [15] (see the PRISMA Checklist).

As a study-level analysis, no ethical approval was needed. The study protocol of this meta-analysis was registered on PROSPERO (CRD420251053635).

### 2.1. Search Strategy

A systematic literature search was carried out in Embase, MEDLINE, and the Cochrane Central Register of Controlled Trials (CENTRAL) from inception to 1 October 2025, for randomized controlled trials (RCTs), prospective, and retrospective cohort studies. The complete search strategy is available online (Supplementary Materials). To complement our search, all references from selected studies were retrieved and manually reviewed. No language restriction was applied.

### 2.2. Eligibility Criteria

Studies were considered eligible if their patient population consisted of adults with diagnosis of PH (any group) and cancer, both solid and hematological. Cohort studies and RCTs were included in the literature search. Prospective and retrospective cohort studies were considered eligible. Each study was required to be available in full text and comprise at least 20 patients. Epidemiological studies based on ICD codes, review articles, and case reports were excluded, as well as those studies presenting a cross-sectional design.

### 2.3. Study Outcomes

We evaluated the prevalence of cancer, both solid and hematological, in patients with PH. Mortality incidence and mortality risk were also evaluated for patients with PH with or without cancer. Specific subanalyses for patients with PH group 4 were also performed. Finally, we evaluated the prevalence of PH and its risk of mortality in patients with cancer.

If a homogeneous definition of PH was not provided, definitions of each included study were used; therefore, in the absence of a clear description of cancer activity across the included studies, the distinction between active and previous cancer was not possible.

### 2.4. Study Selection, Data Extraction, and Quality Assessment

Retrieved studies were imported into Rayyan QCRI management software (https://rayyan.qcri.org, accessed on 1 October 2025). After removal of duplicates, two authors worked independently (FC, AP) for the initial screening of titles and abstracts and then perused the full texts to confirm the eligibility of studies at a second stage. A third author (EV) was consulted to resolve any discordance regarding eligibility of studies. A predefined spreadsheet was created, where two authors (FC, AP) independently extracted data from eligible studies.

A pilot test was performed before the formal initiation of data extraction to ensure coherence. Possible disagreements were resolved by consensus or by discussion with a third review author (EV). Data concerning first author’s name, year of publication and design for all the eligible studies, as well as population characteristics and outcomes were extracted. Two authors (FC, AP) independently evaluated the quality of the studies, according to the Newcastle–Ottawa Scale (NOS) for cohort studies [16].

### 2.5. Strategy for Data Synthesis

Pooled estimates with their 95% confidence intervals (CIs) were obtained using a random-effects model based on the inverse variance approach, with the DerSimonian–Laird method applied to estimate t^2^. Similarly, pooled odds ratios (ORs) and corresponding 95% CIs were derived through a random-effects model using the Mantel–Haenszel method. The Sidik–Jonkman approach was adopted for t^2^ estimation, and the Hartung–Knapp adjustment was implemented for the random-effects model. When studies included zero cell counts, a continuity correction of 0.5 was applied. Heterogeneity was evaluated according to the following I^2^ thresholds: (i) 0–40% suggesting minimal or no heterogeneity, (ii) 30–60% indicating moderate heterogeneity, (iii) 50–90% reflecting substantial heterogeneity, and (iv) 75–100% representing considerable heterogeneity. Nonetheless, the interpretation of I^2^ values also considered the effect size direction, magnitude, and strength of evidence for heterogeneity. Publication bias was explored by visual inspection of funnel plots of logit-transformed proportions against their standard errors, with funnel plot asymmetry evaluated using Egger’s test. Statistical analysis was conducted in RStudio (version 1.2.5001) using the “meta” and “forest” packages.

## 3. Results

### 3.1. Systematic Review of the Literature

**Figure 1:**
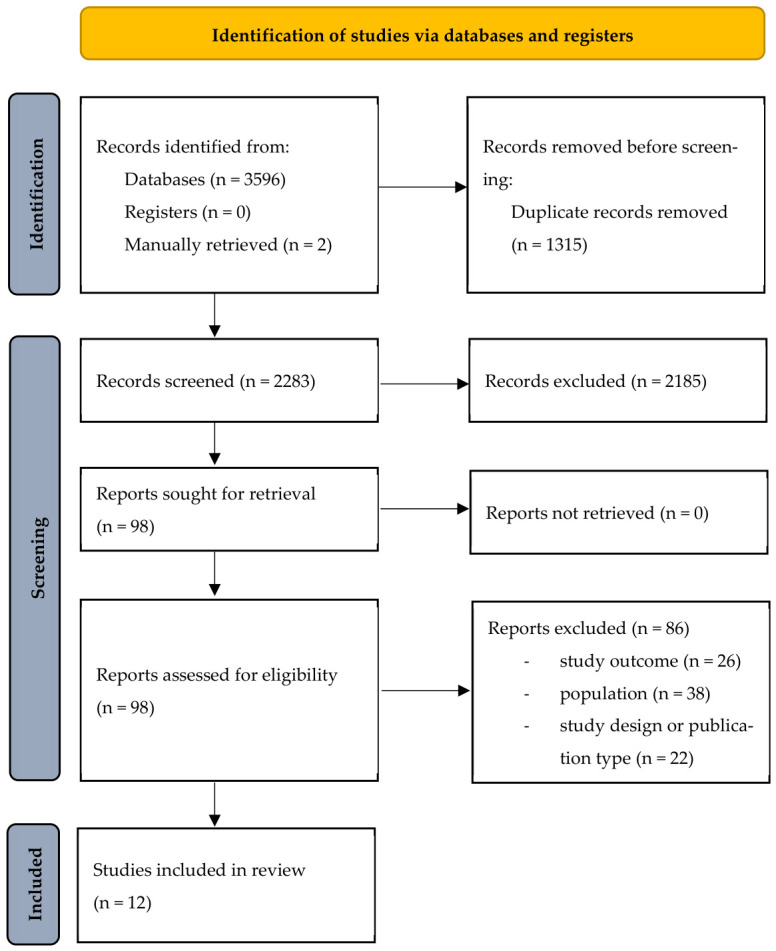
Study selection process according to the search strategy.

We identified 3598 potential studies and eventually included in the systematic review and quantitative analysis a total of 12 studies. The process of study selection is described in Figure 1. All the included studies were observational (*n* = 12; 4402 patients), with eight retrospective (2478 patients) and four prospective studies (1924 patients). The characteristics of the studies included in the meta-analysis are listed in Table 1. The mean age of patients in individual studies ranged from 57 to 73 years. A total of 1927 patients had cancer, and 3111 had PH (group 1 = 34, group 3 = 9, group 4 = 2065, unspecified = 1003). Other key study characteristics used in the individual studies are summarized in the Supplementary Materials (Tables S1 and S2). The quality assessment of the included studies is shown in Table S3. We graded no study as low-quality, seven studies as moderate-quality, and five as high-quality studies. Finally, funnel plots for the study outcomes are shown in Figure S1.

### 3.2. Study Outcomes

#### 3.2.1. Cancer Prevalence in Patients with PH

**Figure 2:**
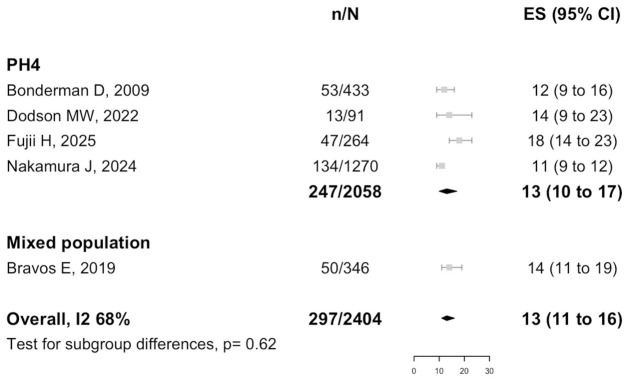
Prevalence of cancer in patients with PH. Included studies: Bonderman D, 2009 [18]; Dodson MW, 2022 [19]; Fujii H, 2025 [20]; Nakamura J, 2024 [8]; Bravos E, 2019 [7].

Data on the prevalence of cancer in patients with PH were available from five studies (*n* = 2404), as shown in Figure 2. The prevalence among the patients with PH group 4 was 13% (95% CI: 10–17%), whereas the prevalence among those with any PH group was 14% (95% CI: 11–19%), with an overall prevalence of 13% (95% CI: 11–16%; I^2^ = 68%; t^2^ = 0.05).

#### 3.2.2. All-Cause Mortality Incidence in Patients with PH

**Figure 3:**
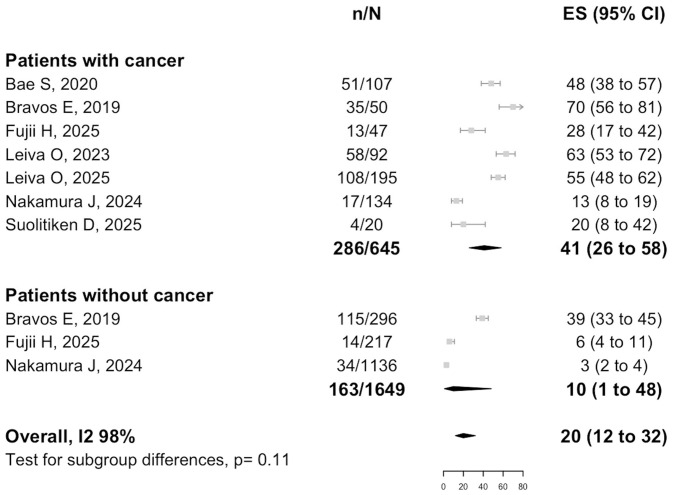
Incidence of all-cause mortality in patients with PH with or without cancer. Included studies: Bae S, 2020 [17]; Bravos E, 2019 [7]; Fujii H, 2025 [20]; Leiva O, 2023 [21]; Leiva O, 2025 [22]; Nakamura J, 2024 [8]; Soulitiken D, 2025 [26].

Data on the incidence of all-cause mortality in patients with any PH group were available from seven studies (*n* = 2294), as shown in Figure 3. The incidence among the patients with PH and cancer was 41% (95% CI: 26–58%), whereas the incidence among those with PH without cancer was 10% (95% CI: 1–48%), with an overall incidence of 20% (95% CI: 12–32%; I^2^ = 98%; t^2^ = 1.11).

Finally, data on the incidence of all-cause mortality in patients with PH group 4 were available from 2 studies (*n* = 1534), as shown in Figure S2. The incidence among the patients with PH group 4 and cancer was 19% (95% CI: 8–37%), whereas the incidence among those with PH group 4 without cancer was 4% (95% CI: 2–9%), significantly lower (*p* for difference: < 0.01). The overall incidence of the two groups was 9% (95% CI: 3–23%; I^2^ = 95%; t^2^ = 1.13).

#### 3.2.3. All-Cause Mortality Risk in Patients with PH

**Figure 4:**
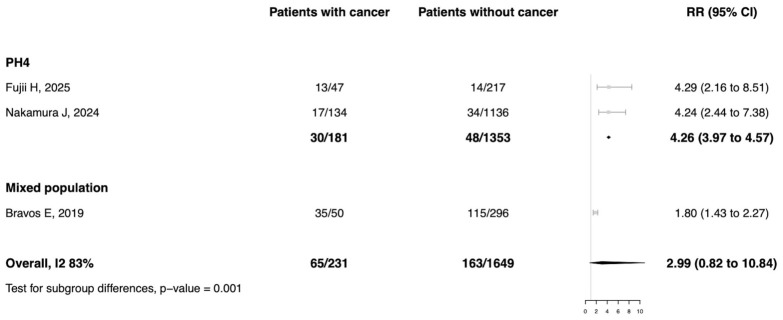
Risk of all-cause mortality in patients with PH with vs. without cancer. Included studies: Fujii H, 2025 [20]; Nakamura J, 2024 [8]; Bravos E, 2019 [7].

Data to assess the risk of all-cause mortality in patients with PH and cancer were available from three studies (*n* = 1880), as shown in Figure 4. Patients with any PH group and cancer showed a significantly higher mortality risk compared to those without cancer (RR: 1.80; 95% CI: 1.43–2.27). Results remained consistent when studies including patients with PH group 4 were analyzed separately (RR: 4.26; 95% CI: 3.97–4.57; I^2^ = 0%; t^2^ < 0.01).

#### 3.2.4. PH in Patients with Cancer and PH-Related Mortality in Patients with Cancer

Data on the prevalence of any PH group in patients with cancer were available from seven studies (*n* = 1587), as shown in Figure S3, with a proportion of 22% (95% CI: 15–31%; I^2^ = 93%; t^2^ = 0.37).

Finally, data to assess the risk of all-cause mortality in patients with cancer and any PH group were available from four studies (*n* = 1272), as shown in Figure S4. Patients with cancer and any PH group showed a trend towards higher mortality risk compared to those without PH (RR: 2.27; 95% CI: 0.76–6.83; I^2^ = 63%; t^2^ = 0.45).

### 3.3. Publication Bias

The visual assessment of the funnel plot did not identify a publication bias for mortality (t = −0.45; df = 8; *p*-value = 0.67). Due to the limited number of studies, it was not possible to perform Egger’s test for the other outcomes.

## 4. Discussion

The results of this study provide the first comprehensive summary of the current evidence on the association between PH and cancer, with a specific focus on mortality. According to our findings, the prevalence of cancer among patients with PH exceeds 10%. The epidemiological study of Roderburg et al. found a significant HR of 1.3 (up to 1.5 in individuals aged 80 years and over) for the association between ICD-diagnosed PH and cancer in 11,109 patients with PH [27]. In our study, patients diagnosed with CTEPH and cancer exhibited a significantly higher mortality incidence compared to those with CTEPH alone. This finding further strengthens the consistency of mortality risk evaluation, both for the overall cohort of PH patients and the specific subgroup of CTEPH, by comparing those with an oncological diagnosis to those without. These results reinforce the hypothesis that cancer represents an independent risk factor for mortality in patients with CTEPH [28,29,30,31], thereby supporting the assumption that these individuals may represent a distinct population with unique characteristics and may therefore benefit not only from usual screening strategies but probably from a more aggressive approach and monitoring to seek for the presence of cancer. In addition, the development of PH in oncologic patients should be more carefully monitored, as they may benefit from early diagnosis and the timely initiation of specific therapies.

With regard to malignancy, it was not possible to perform dedicated subgroup analyses since information concerning cancer type, stage, activity, and treatment was available from only a limited number of studies, with very heterogenous characteristics. Nevertheless, prior epidemiological studies tried to address this issue and, despite inherent limitations related to the use of ICD-based definitions, malignancies that appeared to be most frequently associated with PH were airways and dermatologic tumors [27]. A small study investigated 346 patients with pre-capillary PH without history of current malignancy or previous chemotherapy, detecting an overall cancer incidence of nearly 15%, with lung cancer accounting for 36% of cases, gastrointestinal malignancies for 32%, and hematologic neoplasms for 12% [7]. These findings are only partially consistent with the concept of pulmonary tumor embolism, defined by the obstruction of pulmonary capillaries by tumor cells, without overt pulmonary metastases mostly reported in patients with adenocarcinomas, including those of liver, kidney, stomach, and urinary bladder [32,33]. This underscores that the relationship between PH and cancer remains incompletely understood, highlighting the need for further research to clarify the pathophysiological mechanisms connecting these two conditions.

Finally, our study highlighted a high prevalence of PH among patients with hematological malignancies, particularly MPNs, exceeding 20%. Moreover, the presence of PH in this population appears to be associated with a trend toward increased risk of mortality. The reasons underlying such a high prevalence of PH in this population are multiple and only partially clarified, such as splenomegaly that can lead to porto-pulmonary hypertension and extramedullary hematopoiesis that predisposes to high-output heart failure. Patients with MPNs are at increased risk of developing both pre-capillary (group 4 and 5) and post-capillary (group 2) PH, given the high prevalence of heart failure [13,21,34,35]. Particular attention should be given to CTEPH, which appears to be particularly associated with polycythemia vera and essential thrombocythemia [13]. Patients with MPNs who develop PH appear to be at increased risk of mortality due to a higher likelihood of progression to myelofibrosis and leukemic transformation [21,36,37]. In addition, PH in this setting seems to be associated with a globally increased risk of major adverse cardiovascular events (MACEs). In this population, the management of PH is supported by limited evidence and is often challenging to implement. Moreover, PH may restrict the use of standard oncologic therapies, while certain cancer treatments themselves can contribute to the development or worsening of PH.

Our study presents several limitations that need to be addressed. First, the design of the included studies, which are exclusively observational and mostly single-center and retrospective, may have influenced the overall results: in particular, the high prevalence of cancer in patients with PH may reflect a selection bias related to the closer medical surveillance of this population; second, in the vast majority of the included studies, the diagnosis of PH relied on echocardiography rather than RHC, thus reflecting only a probabilistic assessment of PH and potentially leading to misclassification and overestimate of PH itself; third, only a limited number of studies provided information on cancer characteristics, differentiating between active and inactive cancers, which precluded the possibility of performing dedicated subgroup analyses with distinct results; fourth, due to the paucity of studies reporting data on different PH groups, specific subanalyses were only possible for CTEPH: this may limit the generalizability of our findings for such a heterogeneous disease. On the other hand, based on a rigorous methodology and strict inclusion criteria, our study is the first systematic review and meta-analysis to synthesize all available evidence on the association between cancer and PH, providing the most recent and complete knowledge on a topic of increasing clinical interest.

Given these considerations, how can robust and conclusive evidence in this field be generated in the future? Reliable non-invasive methods are needed to diagnose PH, as many patients never undergo RHC: in the study by Ferrari et al. the overall incidence of PH in patients with MPNs was 35%, which dropped to 7.2% when RHC was performed [38]. In addition, standardized definitions of past and current malignancies, including type and staging, are essential. Finally, a comprehensive work-up is critical to identify the primary cause of PH, taking into account comorbidities and cancer therapies, in order to guide timely and appropriate management.

In conclusion, our findings suggest a potential association between pulmonary hypertension, particularly CTEPH, and cancer, which could represent an additional risk factor for mortality. These results are hypothesis-generating and warrant further investigation to establish a definitive association. These patients may therefore constitute a high-risk population who could benefit from closer surveillance for early cancer detection. Moreover, individuals with hematologic malignancies, especially MPNs, show a high prevalence of PH and a predisposition to adverse outcomes, highlighting the necessity of a multidisciplinary approach to manage both the malignancy and its associated complications. Large, well-designed prospective studies are needed to provide definitive evidence, refine risk stratification, and guide the multidisciplinary management of these patients.

## Figures and Tables

**Table 1:** Characteristics of the included studies.

Author, Year	*N*	Cancer	Type of PH	Study Design	Women, %	Age, Years (Mean ± SD)
Bae S, 2020 [17]	390	390	107 PH patients	Retrospective cohort study	PH: 48.6% non-PH: 43.4%	PH: 69 ± 8 non-PH: 66 ± 10
Bonderman D, 2009 [18]	687	64	433 PH patients type 4	Retrospective cohort study	-	-
Bravos E, 2019 [7]	346	50	34 PH patients type 1 9 PH patients type 3 7 patients type 4	Prospective cohort study	-	-
Dodson MW, 2022 [19]	248	45	91 PH patients type 4	Prospective cohort study	-	-
Fujii H, 2025 [20]	264	47	264 PH patients type 4	Retrospective cohort study	74.6%	67 ± 12
Leiva O, 2023 [21]	197	197	92 PH patients	Retrospective cohort study	PH: 53.2% non-PH: 67.6%	PH: 73 non-PH: 71
Leiva O, 2025 [22]	555	555	195 PH patients	Retrospective cohort study	PH: 50.2% non-PH: 52.2%	PH: 71 ± 7 non-PH: 66 ± 7
Lopez-Mattei J, 2020 [23]	143	143	20 PH patients	Retrospective cohort study	PH: 25% non-PH: 48.8%	PH: 72 ± 7 non-PH: 67 ± 10
Mattar MM, 2016 [24]	60	60	7 PH patients	Prospective cohort study	-	-
Nakamura J, 2024 [8]	1270	134	1270 PH patients type 4	Prospective cohort study	70.7%	67 ± 13
Song IC, 2021 [25]	112	112	12 PH patients	Retrospective cohort study	PH: 41.6% non-PH: 41%	PH: 66 non-PH: 57
Suolitiken D, 2025 [26]	130	130	20 PH patients	Retrospective cohort study	PH: 65% non-PH: 40.9%	PH: 66 ± 12 non-PH: 58 ± 16

PH: pulmonary hypertension.

## Data Availability

The original contributions presented in this study are included in the article. Further inquiries can be directed to the corresponding author.

## Supplementary Materials

The following supporting information can be downloaded at: https://www.mdpi.com/article/10.3390/biomedicines14040876/s1, PRISMA Checklist; Figure S1: Funnel plots; Figure S2: Incidence of all-cause mortality in patients with PH group 4 with or without cancer; Figure S3: Prevalence of PH in patients with cancer; Figure S4: Risk of all-cause mortality in patients with cancer and with vs. without PH; Table S1: Countries and years of inclusion; Table S2: Definitions of outcomes and duration of follow-up; Table S3: The risk of bias assessment of the included studies; the literature search strategy. Studies cited in the Supplementary Materials: Bae S, 2020 [17]; Bonderman D, 2009 [18]; Bravos E, 2019 [7]; Dodson MW, 2022 [19]; Fujii H, 2025 [20]; Leiva O, 2023 [21]; Leiva O, 2025 [22]; Lopez-Mattei J, 2020 [23]; Mattar MM, 2016 [24]; Nakamura J, 2024 [8]; Song IC, 2021 [25]; Suolitiken D, 2025 [26].

## References

[1] Simonneau G., Montani D., Celermajer D.S., Denton C.P., Gatzoulis M.A., Krowka M., Williams P.G., Souza R. (2019). Haemodynamic definitions and updated clinical classification of pulmonary hypertension. Eur. Respir. J..

[2] Humbert M., Kovacs G., Hoeper M.M., Badagliacca R., Berger R.M., Brida M., Carlsen J., Coats A.J., Escribano-Subias P., Ferrari P. (2023). 2022 ESC/ERS Guidelines for the diagnosis and treatment of pulmonary hypertension. Eur. Respir. J..

[3] Moslehi J.J., Deininger M. (2015). Tyrosine kinase inhibitor-associated cardiovascular toxicity in chronic myeloid leukemia. J. Clin. Oncol..

[4] Moslehi J.J. (2016). Cardiovascular Toxic Effects of Targeted Cancer Therapies. N. Engl. J. Med..

[5] Barber M.C., Mauro M.J., Moslehi J. (2017). Cardiovascular care of patients with chronic myeloid leukemia (CML) on tyrosine kinase inhibitor (TKI) therapy. Hematol. Am. Soc. Hematol. Educ. Program.

[6] Lyon A.R., López-Fernández T., Couch L.S., Asteggiano R., Aznar M.C., Bergler-Klein J., Boriani G., Cardinale D., Cordoba R., Cosyns B. (2022). 2022 ESC Guidelines on cardio-oncology developed in collaboration with the European Hematology Association (EHA), the European Society for Therapeutic Radiology and Oncology (ESTRO) and the International Cardio-Oncology Society (IC-OS). Eur. Heart J..

[7] Bravos E., Cottin V., Dauphin C., Bouvaist H., Traclet J., Trésorier R., Margelidon-Cozzolino V., Bezzeghoud S., Ahmad K., Accassat S. (2019). Cancer incidence in patients with pre-capillary pulmonary hypertension. J. Heart Lung Transplant..

[8] Nakamura J., Tsujino I., Masaki K., Hosokawa K., Funakoshi K., Taniguchi Y., Adachi S., Inami T., Yamashita J., Ogino H. (2025). Cancer as an independent mortality risk in chronic thromboembolic pulmonary hypertension. J. Heart Lung Transplant..

[9] Lang I.M., Pesavento R., Bonderman D., Yuan J.X.-J. (2013). Risk factors and basic mechanisms of chronic thromboembolic pulmonary hypertension: A current understanding. Eur. Respir. J..

[10] Kerr K.M., Elliott C.G., Chin K., Benza R.L., Channick R.N., Davis R.D., He F., LaCroix A., Madani M.M., McLaughlin V.V. (2021). Results From the United States Chronic Thromboembolic Pulmonary Hypertension Registry: Enrollment Characteristics and 1-Year Follow-up. Chest.

[11] Lichtblau M., Lador F., Lechartier B., Ellegast J.M., Pohle S., Preiss H., Genecand L., Wick C., Ulrich S., Reimann L. (2025). Pulmonary Hypertension in the Field of Hematological and Oncological Diseases. Respiration.

[12] Price L.C., Seckl M.J., Dorfmüller P., Wort S.J. (2019). Tumoral pulmonary hypertension. Eur. Respir. Rev..

[13] Montani D., Thoré P., Mignard X., Jaïs X., Boucly A., Jevnikar M., Seferian A., Jutant E.-M., Cottin V., Fadel E. (2023). Clinical Phenotype and Outcomes of Pulmonary Hypertension Associated with Myeloproliferative Neoplasms A Population-based Study. Am. J. Respir. Crit. Care Med..

[14] Leiva O., Beaty W., Soo S., Agarwal M.A., Yang E.H. (2024). Cancer Therapy-Associated Pulmonary Hypertension and Right Ventricular Dysfunction: Etiologies and Prognostic Implications. Rev. Cardiovasc. Med..

[15] Moher D., Liberati A., Tetzlaff J., Altman D.G., PRISMA Group (2019). Preferred reporting Items for systematic reviews and meta-analyses: The PRISMA statement. PLoS Med..

[16] Wells G.A., Shea B.J., O’Connell D., Peterson J., Welch V., Losos M., Tugwell P. (2014). The Newcastle-Ottawa Scale (NOS) for assessing the quality of non-randomised studies in meta-analyses. Appl. Eng. Agric..

[17] Bae S., Kim K.H., Yoon H.J., Kim H.Y., Park H., Cho J.Y., Kim M.C., Kim Y., Hong Y.J., Park H.W. (2020). Clinical impact of echocardiography-defined pulmonary hypertension on the clinical outcome in patients with multiple myeloma. Medicine.

[18] Bonderman D., Wilkens H., Wakounig S., Schäfers H.-J., Jansa P., Lindner J., Simkova I., Martischnig A.M., Dudczak J., Sadushi R. (2009). Risk factors for chronic thromboembolic pulmonary hypertension. Eur. Respir. J..

[19] Dodson M.W., Cirulis M.M., Li H., Yue Z., Brown L.M., Elliott C.G. (2022). Frequency of Thrombotic Risk Factors in Patients with Chronic Thromboembolic Pulmonary Hypertension and Acute Pulmonary Embolism: A Case-Control Study. Clin. Appl. Thromb./Hemost..

[20] Fujii H., Taniguchi Y., Tamura Y., Sakamoto M., Yoneda S., Yanaka K., Emoto N., Hirata K.-I., Otake H. (2025). Association between the prognosis and comorbidity of active cancer in chronic thromboembolic pulmonary hypertension. BMC Pulm. Med..

[21] Leiva O., Ren S., Neuberg D., Bhatt A., Jenkins A., Rosovsky R., Leaf R.K., Goodarzi K., Hobbs G. (2023). Pulmonary hypertension is associated with poor cardiovascular and hematologic outcomes in patients with myeloproliferative neoplasms and cardiovascular disease. Int. J. Hematol..

[22] Leiva O., Soo S., Liu O., Smilowitz N.R., Reynolds H., Shah B., Bernard S., How J., Lee M.H., Hobbs G. (2025). Characterization and prognostic implication of pulmonary hypertension among patients with myeloproliferative neoplasms. Haematologica.

[23] Lopez-Mattei J., Verstovsek S., Fellman B., Iliescu C., Bhatti K., Hassan S.A., Kim P., Gray B.A., Palaskas N.L., Grosu H.B. (2020). Prevalence of pulmonary hypertension in myelofibrosis. Ann. Hematol..

[24] Mattar M.M., Morad M.A.K., El Husseiny N.M., Ali N.H., El Demerdash D.M. (2016). Correlation between JAK2 allele burden and pulmonary arterial hypertension and hematological parameters in Philadelphia negative JAK2 positive myeloproliferative neoplasms. An Egyptian experience. Ann. Hematol..

[25] Suolitiken D., Han X., Feng C., Wang Lee H.-J., Yun H.-J., Sun B.J., Park J.-H., Jeong J.-O., Jo D.-Y. (2021). Pulmonary hypertension in patients with chronic myeloid leukemia. Medicine.

[26] Suolitiken D., Han X., Feng C., Wang Y. (2025). Transformed to myelofibrosis is a risk factor for pulmonary hypertension in Philadelphia chromosome-negative myeloproliferative neoplasms. Ann. Hematol..

[27] Roderburg C., Loosen S.H., Hippe H., Luedde T., Kostev K., Luedde M. (2022). Pulmonary hypertension is associated with an increased incidence of cancer diagnoses. Pulm. Circ..

[28] Delcroix M., Lang I., Pepke-Zaba J., Jansa P., D’Armini A.M., Snijder R., Bresser P., Torbicki A., Mellemkjaer S., Lewczuk J. (2016). Long-Term Outcome of Patients with Chronic Thromboembolic Pulmonary Hypertension: Results from an International Prospective Registry. Circulation.

[29] Saouti N., de Man F., Westerhof N., Boonstra A., Twisk J., Postmus P.E., Noordegraaf A.V. (2009). Predictors of mortality in inoperable chronic thromboembolic pulmonary hypertension. Respir. Med..

[30] Hosokawa K., Abe K., Funakoshi K., Tamura Y., Nakashima N., Todaka K., Taniguchi Y., Inami T., Adachi S., Tsujino I. (2023). Long-term outcome of chronic thromboembolic pulmonary hypertension using direct oral anticoagulants and warfarin: A Japanese prospective cohort study. J. Thromb. Haemost..

[31] Nakamura J., Tsujino I., Shima H., Nakaya T., Sugimoto A., Sato T., Watanabe T., Ohira H., Suzuki M., Yokota I. (2023). Impact of cancer on the prevalence, management, and outcome of patients with chronic thromboembolic pulmonary hypertension. J. Thromb. Thrombolysis.

[32] Kane G.C., Karon B.L., Mahoney D.W., Redfield M.M., Roger V.L., Burnett J.C., Jacobsen S.J., Rodeheffer R.J. (2011). Progression of Left Ventricular Diastolic Dysfunction and Risk of Heart Failure. JAMA.

[33] Roberts K.E., Hamele-Bena D., Saqi A., Stein C., Cole R.P. (2003). Pulmonary tumor embolism: A review of the literature. Am. J. Med..

[34] Leiva O., Hobbs G., Ravid K., Libby P. (2022). Cardiovascular Disease in Myeloproliferative Neoplasms: *JACC: CardioOncology* State-of-the-Art Review. J. Am. Coll. Cardiol. CardioOnc..

[35] Leiva O., Garcia B.D., Hobbs G. (2023). Pulmonary Hypertension in Myeloproliferative Neoplasms: New Insights and Unexplored Horizons. Am. J. Respir. Crit. Care Med..

[36] Kim J., Krichevsky S., Xie L., Palumbo M.C., Rodriguez-Diego S., Yum B., Brouwer L., Silver R.T., Schafer A.I., Ritchie E.K. (2019). Incremental Utility of Right Ventricular Dysfunction in Patients With Myeloproliferative Neoplasm-Associated Pulmonary Hypertension. J. Am. Soc. Echocardiogr..

[37] Yaylali Y.T., Yilmaz S., Akgun-Cagliyan G., Kilic O., Kaya E., Senol H., Ozen F. (2020). Association of Disease Subtype and Duration with Echocardiographic Evidence of Pulmonary Hypertension in Myeloproliferative Neoplasm. Med. Princ. Pract..

[38] Ferrari A., Scandura J., Masciulli A., Krichevsky S., Gavazzi A., Barbui T. (2021). Prevalence and risk factors for Pulmonary Hypertension associated with chronic Myeloproliferative Neoplasms. Eur. J. Haematol..

