# Supplementary material for: Exploring the Association Between Pulmonary Hypertension and Cancer: A Systematic Review and Meta-Analysis
Source: Biomedicines. 2026 Apr 11;14(4):876. doi: 10.3390/biomedicines14040876 (PMC13114216; doi:10.3390/biomedicines14040876)
Supplement: Supplementary file 1 [file biomedicines-14-00876-s001.zip › biomedicines-4191590-PRISMA checklist.pdf]

PRISMA 2020 Checklist

Section and Topic	Item #	Checklist item	Location where item is reported
**TITLE**			
Title	1	Exploring the association between pulmonary hypertension and cancer: A systematic review and meta-analysis.	Title, Page 1
**ABSTRACT**			
Abstract	2	According to the PRISMA 2020 for Abstracts checklist, all the items are reported below.	Abstract, Page 1
**INTRODUCTION**			
Rationale	3	Cancer and pulmonary circulation disorders represent increasingly intersecting clinical entities. The prevalence of malignancy in patients with pulmonary hypertension, particularly those with chronic thromboembolic pulmonary hypertension, is higher than in the general population. Moreover, cancer and antineoplastic therapies have been implicated in the development of pulmonary hypertension through multiple mechanisms.	Section 1, Page 2
Objectives	4	The main focuses were to assess the prevalence of cancer in patients with both pulmonary hypertension (any group) and chronic thromboembolic pulmonary hypertension, the prevalence of pulmonary hypertension associated with myeloproliferative neoplasms, and their impact on patients' mortality.	Section 1, Page 2
**METHODS**			
Eligibility criteria	5	Studies were considered eligible if their patient population consisted of adults with diagnosis of pulmonary hypertension (any group) and cancer, both solid and haematological. Cohort studies and RCTs were included in the literature search. Prospective and retrospective cohort studies were considered eligible. Each study was required to be available in full text and comprise at least 20 patients. Epidemiological studies based on ICD codes, review articles, and case reports were excluded, as well as those studies presenting a cross-sectional design.	Section 2.2, Page 3
Information sources	6	A systematic literature search was carried out in Embase, MEDLINE, and the Cochrane Central Register of Controlled Trials (CENTRAL) from inception to October 1st 2025, for RCTs, prospective, and retrospective cohort studies.	Section 2.1, Page 3
Search strategy	7	1 exp Hypertension, Pulmonary/<br>2 exp Pulmonary Arterial Hypertension/<br>3 exp Pulmonary Vascular Diseases/<br>4 (pulmon* adj3 hypertens*).mp.<br>5 (pulmonary adj3 hypertens*).mp.<br>6 (pulmonary adj3 arterial adj3 hypertens*).mp.<br>7 (PAH or "pulmonary arterial hypertens*").mp.<br>8 (CTEPH or "chronic thromboembolic pulmonary hypertens*" or "chronic thromboembolic PH").mp.<br>9 (PVOD or "pulmonary veno-occlusiv*").mp.<br>10 (portopulmonary or "portopulmonary hypertens*").mp.<br>11 or/1-10<br>12 exp Neoplasms/<br>13 (neoplasm* or neoplas* or tumor* or tumour* or malignan* or cancer* or carcinoma*).mp.<br>14 exp Incidence/ or exp Epidemiology/ or exp Risk/<br>15 (inciden* or incidence rate* or "incidence rate" or prevalen* or "population-based" or "cohort" or "cohort stud*" or longitudinal* or "epidemiolog*").mp.<br>16 12 or 13<br>17 14 or 15<br>18 11 and 16 and 17<br>19 limit 18 to full text<br>20 limit 19 to human<br>21 limit 20 to humans	Supplementary Material
Selection process	8	After removal of duplicates, two authors worked independently for the initial screening of titles and abstracts and then perused the full texts to confirm the eligibility of studies at a second stage. A third author was consulted to resolve any discordance regarding eligibility of studies.	Section 2.4, Page 3
Data collection process	9	A predefined spreadsheet was created, where two authors independently extracted data from eligible studies. A pilot test was performed before the formal initiation of data extraction to ensure coherence. Possible disagreements were resolved by consensus or by discussion with	Section 2.4, Page 3

Section and Topic	Item #	Checklist item	Location where item is reported
		a third review author**.**	
Data items	10a	We evaluated the prevalence of cancer, both solid and haematological, in patients with pulmonary hypertension. Mortality incidence and mortality risk were also evaluated for patients with pulmonary hypertension with or without cancer. Specific subanalysis for patients with pulmonary hypertension group 4 were also performed. Finally, we evaluated the prevalence of pulmonary hypertension and its risk of mortality in patients with cancer.	Section 2.3, Page 3
	10b	If a homogeneous definition of pulmonary hypertension was not provided, definitions of each included study were used; therefore, in the absence of a clear description of cancer activity across the included studies, the distinction between active and previous cancer was not possible.	Section 2.3, Page 3
Study risk of bias assessment	11	Two authors independently evaluated the quality of the studies, according to the Newcastle–Ottawa Scale (NOS) for cohort studies.	Section 2.4, Page 3
Effect measures	12	The outcomes were express in proportion (%) or risk ratio (RR).	Section 2.5, Page 3
**RESULTS**			
Study selection	13a	We identified 3598 potential studies and eventually included in the systematic review and quantitative analysis a total of 12 studies.	Section 3.1, Page 4
	13b	86 studies appeared to meet the inclusion criteria. Nevertheless 26 studies were excluded due to study outcome, 38 studies due to population, and 22 studies due to study design or publication type.	Figure 1, Page 5
Study characteristics	14	See Table 1.	Table 1, Page 4
Risk of bias in studies	15	We graded no study as low-quality, 7 studies as moderate-quality, and 5 as high-quality studies	Section 3.1, Page 4
Results of individual studies	16	See Table S2 (Supplementary Material).	Supplementary Material
**DISCUSSION**			
Discussion	17a	The results of this study provide a comprehensive summary of the current evidence on the association between pulmonary hypertension and cancer and, to the best of our knowledge, it represents the first systematic review and meta-analysis evaluating such an increasingly relevant topic.	Section 4, Page 8
	17b	The heterogeneity of the included studies in terms of patients, outcomes and study design -which is exclusively observational and mostly monocentric and retrospective- may have biased the overall results;	Section 4, Page 9
	17c	Assessing the data only at the study level is an inherent limitation of such meta-analysis as it precludes more specific investigations into how individual patient characteristics may influence the results	Section 4, Page 9
	17d	Our findings suggest a potential association between pulmonary hypertension, particularly chronic thromboembolic pulmonary hypertension, and cancer, representing an additional risk factor for mortality. These patients may therefore constitute a high-risk population who could benefit from closer surveillance for early cancer detection. Moreover, individuals with haematologic malignancies, especially myeloproliferative neoplasms, show a high prevalence of pulmonary hypertension and a predisposition to adverse outcomes, highlighting the necessity of a multidisciplinary approach to manage both the malignancy and its associated complications.	Section 4, Page 9
**OTHER INFORMATION**			
Registration and protocol	18a	The study protocol of this meta-analysis was registered on PROSPERO (CRD420251053635).	Section 4, Page 2
	18b	The study protocol of this meta-analysis was registered on PROSPERO (CRD420251053635).	Section 4, Page 2
	18c	We made no amendments to information provided at registration or in the protocol.	Not applicable
Support	19	This research received no external funding.	Funding, Page 10
Competing	20	There were no competing interests of review authors.	Conflicts of

PRISMA

PRISMA 2020 Checklist

Section and Topic	Item #	Checklist item	Location where item is reported
interests			Interest, Page 10
Availability of data, code and other materials	21	Template data collection forms, data extracted from included studies, data used for all analyses, analytic code are available from the authors upon request.	Not applicable

From: Page MJ, McKenzie JE, Bossuyt PM, Boutron I, Hoffmann TC, Mulrow CD, et al. The PRISMA 2020 statement: an updated guideline for reporting systematic reviews. BMJ 2021;372:n71. doi: 10.1136/bmj.n71. This work is licensed under CC BY 4.0. To view a copy of this license, visit https://creativecommons.org/licenses/by/4.0/
