# Supplementary material for: Exploring the Association Between Pulmonary Hypertension and Cancer: A Systematic Review and Meta-Analysis
Source: Biomedicines. 2026 Apr 11;14(4):876. doi: 10.3390/biomedicines14040876 (PMC13114216; doi:10.3390/biomedicines14040876)
Supplement: Supplementary file 1 [file biomedicines-14-00876-s001.zip › biomedicines-4191590-supplementary.pdf]

Supplementary material

Fig. S1 Funnel plots

A Prevalence of cancer in patients with PH

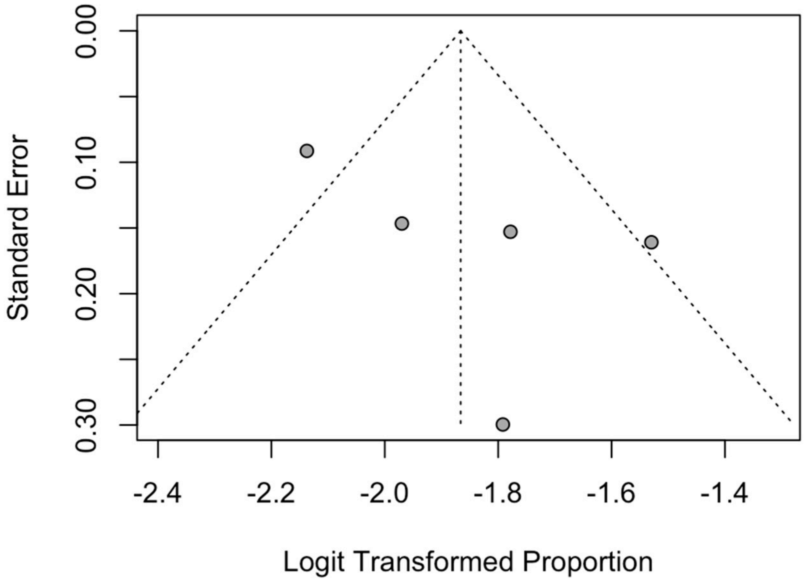

B Incidence of all-cause mortality in patients with PH with or without cancer

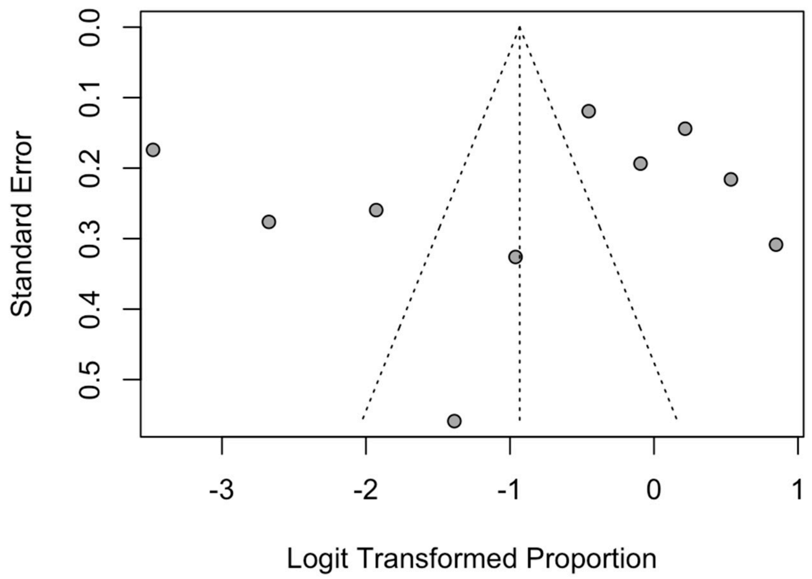

C Incidence of all-cause mortality in patients with PH group 4 with or without cancer

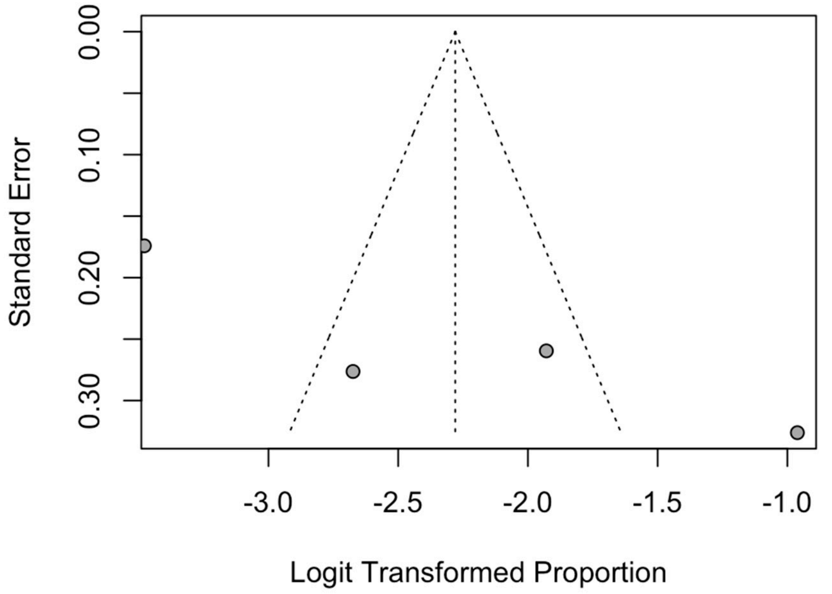

D Risk of all-cause mortality in patients with PH with vs. without cancer

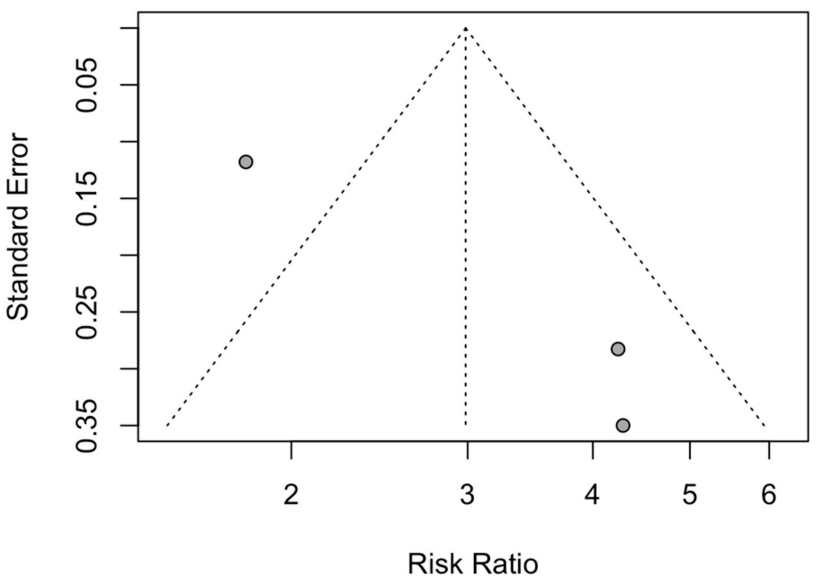

E Prevalence of PH in patients with cancer

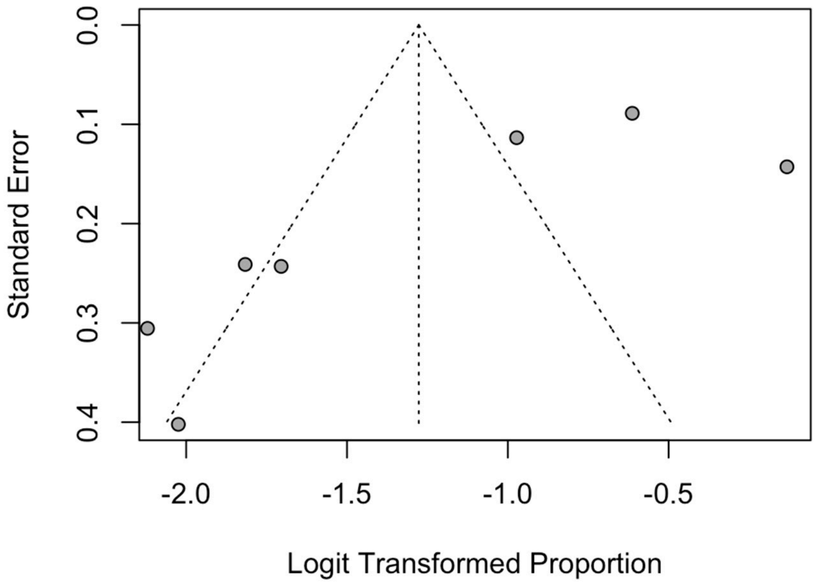

F Risk of all-cause mortality in patients with cancer and with vs. without PH

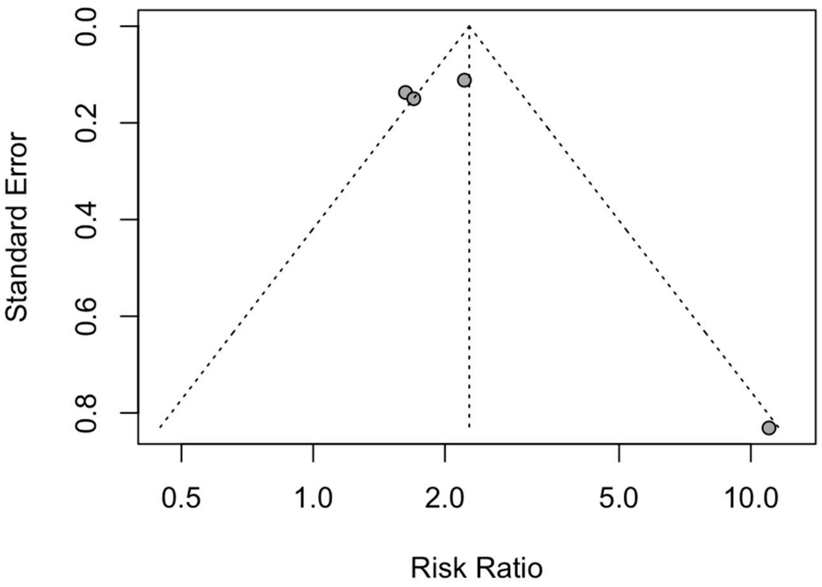

Fig. S2 Incidence of all-cause mortality in patients with PH group 4 with or without cancer

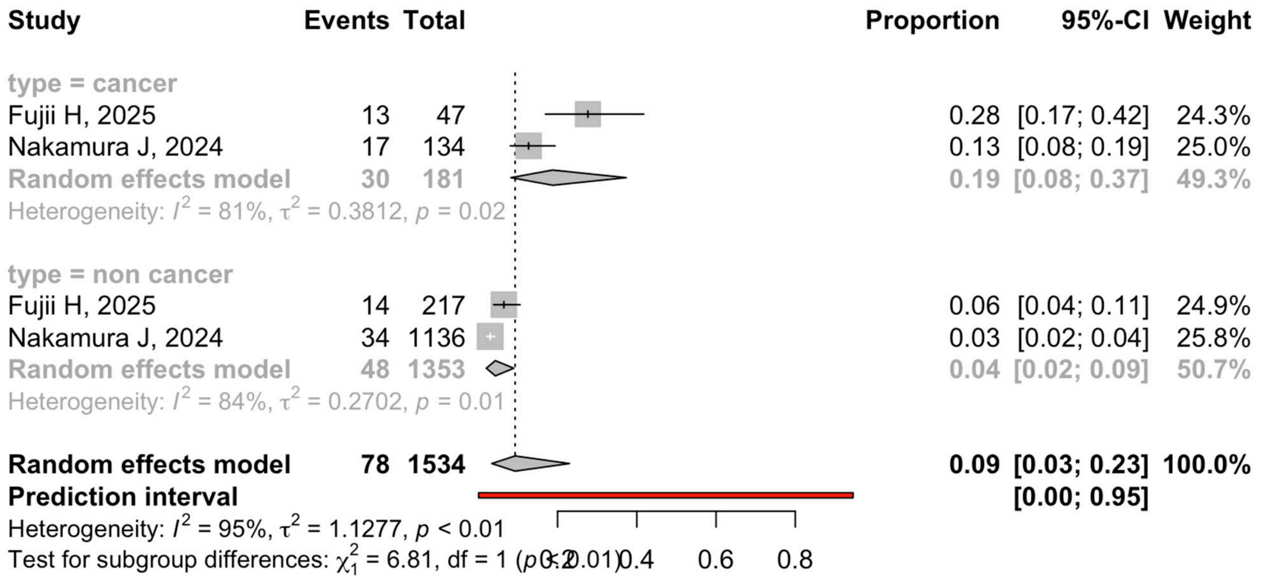

Fig. S3 Prevalence of PH in patients with cancer

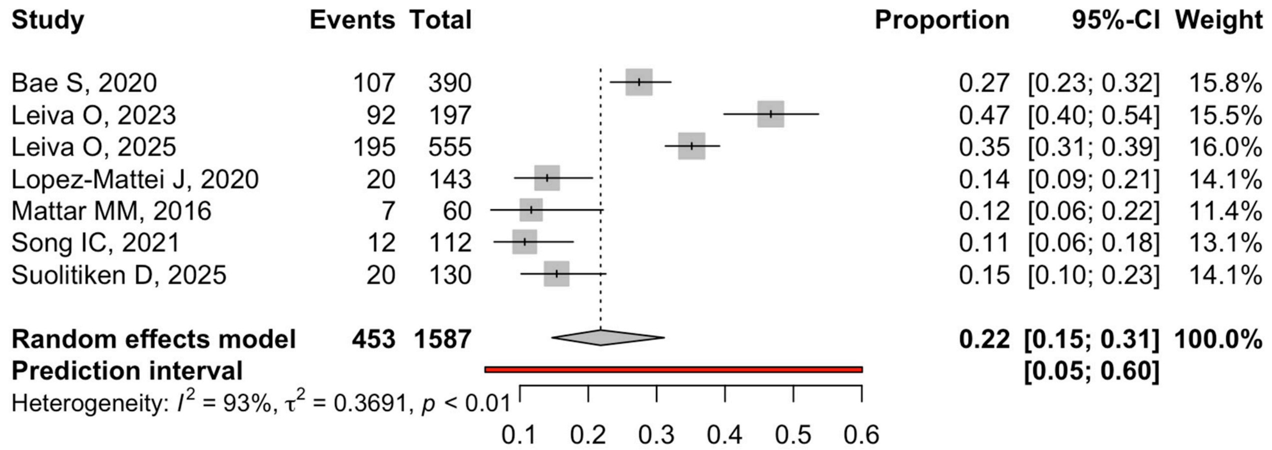

Fig. S4 Risk of all-cause mortality in patients with cancer and with vs. without PH

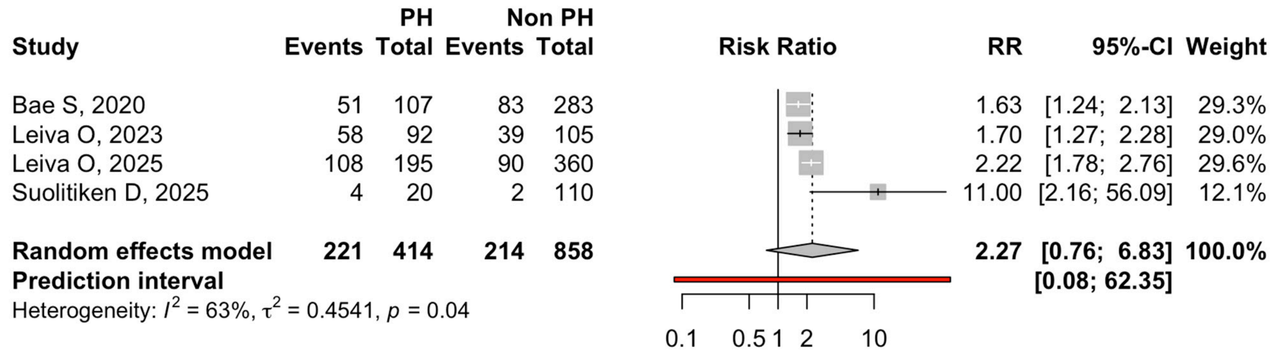

Table S1. Countries and years of inclusion

Author, year	Years of inclusion	Country	Intervention	Collection of data	Multicentre
Bae S, 2020 [1]	2007-2017	Korea	No	Retrospective cohort study	No
Bonderman D, 2009 [2]	1996-2007	Austria	No	Retrospective cohort study	Yes
Bravos E, 2019 [3]	2010-2012	France	No	Prospective cohort study	Yes
Dodson MW, 2022 [4]	2015-2019	USA	No	Prospective cohort study	No
Fujii H, 2025 [5]	2011-2022	Japan	No	Retrospective cohort study	No
Leiva O, 2023 [6]	2000-2020	USA	No	Retrospective cohort study	No
Leiva O, 2025 [7]	2010-2023	USA	No	Retrospective cohort study	Yes
Lopez-Mattei J, 2020 [8]	2015-2017	USA	No	Retrospective cohort study	No
Mattar MM, 2016 [9]	2013-2015	Egypt	No	Prospective cohort study	No
Nakamura J, 2024 [10]	2018-2023	Japan	No	Prospective cohort study	No
Song IC, 2021 [11]	2003-2020	Korea	No	Retrospective cohort study	No
Suolitiken D, 2025 [12]	2020-2024	China	No	Retrospective cohort study	No

Table S2. Definitions of outcomes and duration of follow-up

Author, year	Efficacy outcome results	Follow-up, months (mean)	Author, year	Efficacy outcome results	Follow-up, months (mean)
Bae S, 2020 [1]	All-cause death:<br>PH: 51<br>Non-PH: 83	22.9	Leiva O, 2025 [7]	All-cause death:<br>PH: 108<br>Non-PH: 90	96
Bonderman D, 2009 [2]	-	-	Lopez-Mattei J, 2020 [8]	-	36.6
Bravos E, 2019 [3]	All-cause death:<br>PH: 35<br>Non-PH: 15	26.4	Mattar MM, 2016 [9]	-	-
Dodson MW, 2022 [4]	-	-	Nakamura J, 2024 [10]	All-cause death:<br>PH: 51<br>VTE recurrence:<br>PH: 262	-
Fujii H, 2025 [5]	All-cause death:<br>PH: 27	38	Song IC, 2021 [11]	-	-
Leiva O, 2023 [6]	All-cause death:<br>PH: 58<br>Non-PH: 39	51.2	Suolitiken D, 2025 [12]	All-cause death:<br>PH: 4<br>Non-PH: 2	-

PH: pulmonary hypertension; VTE: venous thromboembolism

Table S3. The risk of bias assessment of the included studies

The Newcastle-Ottawa Scale for assessing the quality of nonrandomized studies in the meta-analysis									
	**Selection**				**Comparability of the cohort**	**Outcome**			**Total score**
**Author, year**	**Representativeness of the exposed cohort**	**Selection of the non-exposed cohort**	**Ascertainment of exposure**	**Outcome not present at baseline**		**Assessment of outcome**	**Enough follow up duration**	**Adequate follow-up**	
Bae S, 2020 [1]	*		*	*	*		*	*	6
Bonderman D, 2009 [2]	*	*	*	*	*	*	*	*	8
Bravos E, 2019 [3]	*	*	*	*		*	*	*	7
Dodson MW, 2022 [4]	*	*	*	*		*	*	*	7
Fujii H, 2025 [5]	*	*	*	*	*	*	*	*	8

Leiva O, 2023 [6]	*		*	*	*		*	*	6
Leiva O, 2025 [7]	*		*	*	*		*	*	6
Lopez-Mattei J, 2020 [8]	*		*	*	*		*	*	6
Mattar MM, 2016 [9]	*		*	*	*		*	*	6
Nakamura J, 2024 [10]	*	*	*	*	*	*	*	*	8
Song IC, 2021 [11]	*		*	*	*		*	*	6
Suolitiken D, 2025 [12]	*		*	*	*		*	*	6

Literature search strategy

1 exp Hypertension, Pulmonary/

2 exp Pulmonary Arterial Hypertension/

3 exp Pulmonary Vascular Diseases/

4 (pulmon* adj3 hypertens*).mp.

5 (pulmonary adj3 hypertens*).mp.

6 (pulmonary adj3 arterial adj3 hypertens*).mp.

7 (PAH or "pulmonary arterial hypertens*").mp.

8 (CTEPH or "chronic thromboembolic pulmonary hypertens*" or "chronic thromboembolic PH").mp.

9 (PVOD or "pulmonary veno-occlusiv*").mp.

10 (portopulmonary or "portopulmonary hypertens*").mp.

11 or/1-10

12 exp Neoplasms/

13 (neoplasm* or neoplas* or tumor* or tumour* or malignan* or cancer* or carcinoma*).mp.

14 exp Incidence/ or exp Epidemiology/ or exp Risk/

15 (inciden* or incidence rate* or "incidence rate" or prevalen* or "population-based" or "cohort" or "cohort stud*" or longitudinal* or "epidemiolog*").mp.

16 12 or 13

17 14 or 15

18 11 and 16 and 17

19 limit 18 to full text

20 limit 19 to human

21 limit 20 to humans

References

1. Bae, S.; Kim, K.H.; Yoon, H.J.; Kim, H.Y.; Park, H.; Cho, J.Y.; Kim, M.C.; Kim, Y.; Hong, Y.J.; Park, H.W.; et al. Clinical impact of echocardiography-defined pulmonary hypertension on the clinical outcome in patients with multiple myeloma. Medicine 2020, 99, e22952. https://doi.org/10.1097/MD.0000000000022952.
2. Bonderman, D.; Wilkens, H.; Wakounig, S.; Schäfers, H.-J.; Jansa, P.; Lindner, J.; Simkova, I.; Martischnig, A.M.; Dudczak, J.; Sadushi, R.; et al. Risk factors for chronic thromboembolic pulmonary hypertension. Eur. Respir. J. 2009, 33, 325–331. https://doi.org/10.1183/09031936.00087608.
3. Bravos, E.; Cottin, V.; Dauphin, C.; Bouvaist, H.; Traclet, J.; Trésorier, R.; Margelidon-Cozzolino, V.; Bezzeghoud, S.; Ahmad, K.; Accassat, S.; et al. Cancer incidence in patients with pre-capillary pulmonary hypertension. J. Heart Lung Transplant. 2019, 38, 778–780. https://doi.org/10.1016/j.healun.2019.03.007.
4. Dodson, M.W.; Cirulis, M.M.; Li, H.; Yue, Z.; Brown, L.M.; Elliott, C.G. Frequency of Thrombotic Risk Factors in Patients with Chronic Thromboembolic Pulmonary Hypertension and Acute Pulmonary Embolism: A Case-Control Study. Clin. Appl. Thromb. /Hemost. 2022, 28, 10760296211073277. https://doi.org/10.1177/10760296211073277.
5. Fujii, H.; Taniguchi, Y.; Tamura, Y.; Sakamoto, M.; Yoneda, S.; Yanaka, K.; Emoto, N.; Hirata, K.-I.; Otake, H. Association between the prognosis and comorbidity of active cancer in chronic thromboembolic pulmonary hypertension. BMC Pulm. Med. 2025, 25, 2. https://doi.org/10.1186/s12890-024-03460-5.
6. Leiva, O.; Ren, S.; Neuberg, D.; Bhatt, A.; Jenkins, A.; Rosovsky, R.; Leaf, R.K.; Goodarzi, K.; Hobbs, G. Pulmonary hypertension is associated with poor cardiovascular and hematologic outcomes in patients with myeloproliferative neoplasms and cardiovascular disease. Int. J. Hematol. 2023, 117, 90–99. https://doi.org/10.1007/s12185-022-03454-1.
7. Leiva, O.; Soo, S.; Liu, O.; Smilowitz, N.R.; Reynolds, H.; Shah, B.; Bernard, S.; How, J.; Lee, M.H.; Hobbs, G. Characterization and prognostic implication of pulmonary hypertension among patients with myeloproliferative neoplasms. Haematologica 2025, 110, 2388–2399. https://doi.org/10.3324/haematol.2025.287497.
8. Lopez-Mattei, J.; Verstovsek, S.; Fellman, B.; Iliescu, C.; Bhatti, K.; Hassan, S.A.; Kim, P.; Gray, B.A.; Palaskas, N.L.; Grosu, H.B.; et al. Prevalence of pulmonary hypertension in myelofibrosis. Ann. Hematol. 2020, 99, 781–789. https://doi.org/10.1007/s00277-020-03962-2.
9. Mattar, M.M.; Morad, M.A.K.; El Husseiny, N.M.; Ali, N.H.; El Demerdash, D.M. Correlation between JAK2 allele burden and pulmonary arterial hypertension and hematological parameters in Philadelphia negative JAK2 positive myeloproliferative neoplasms. An Egyptian experience. Ann. Hematol. 2016, 95, 1611–1616. https://doi.org/10.1007/s00277-016-2765-0.
10. Nakamura, J.; Tsujino, I.; Masaki, K.; Hosokawa, K.; Funakoshi, K.; Taniguchi, Y.; Adachi, S.; Inami, T.; Yamashita, J.; Ogino, H.; et al. Cancer as an independent mortality risk in chronic thromboembolic pulmonary hypertension. J. Heart Lung Transplant. 2025, 44, 339–348. https://doi.org/10.1016/j.healun.2024.10.022.
11. Song, I.-C.; Yeon, S.-H.; Lee, M.-W.; Ryu, H.; Lee, H.-J.; Yun, H.-J.; Sun, B.J.; Park, J.-H.; Jeong, J.-O.; Jo, D.-Y. Pulmonary hypertension in patients with chronic myeloid leukemia. Medicine 2021, 100, e26975. https://doi.org/10.1097/MD.0000000000026975.
12. Suolitiken, D.; Han, X.; Feng, C.; Wang, Y. Transformed to myelofibrosis is a risk factor for pulmonary hypertension in Philadelphia chromosome-negative myeloproliferative neoplasms. Ann. Hematol. 2025, 104, 2279–2285. https://doi.org/10.1007/s00277-025-06334-w.
